# Protocol for a phase 1 homeopathic drug proving trial

**DOI:** 10.1186/1745-6215-11-80

**Published:** 2010-07-22

**Authors:** Michael Teut, Ute Hirschberg, Rainer Luedtke, Cristoph Schnegg, Joern Dahler, Henning Albrecht, Claudia M Witt

**Affiliations:** 1 Institute for Social Medicine, Epidemiology and Health Economics; Charité University Medical Center, Berlin, Germany; 2 Karl and Veronica Carstens Foundation, Essen, Germany

## Abstract

**Background:**

This study protocol adapts the traditional homeopathic drug proving methodology to a modern clinical trial design.

**Method:**

Multi-centre, randomised, double-blind, placebo-controlled phase 1 trial with 30 healthy volunteers. The study consists of a seven day run-in period, a five day intervention period and a 16 day post-intervention observation period. Subjects, investigators and the statisticians are blinded from the allocation to the study arm and from the identity of the homeopathic drug. The intervention is a highly diluted homeopathic drug (potency C12 = 10^24^), Dose: 5 globules taken 5 times per day over a maximum period of 5 days. The placebo consists of an optically identical carrier substance (sucrose globules). Subjects document the symptoms they experience in a semi-structured online diary. The primary outcome parameter is the number of specific symptoms that characterise the intervention compared to the placebo after a period of three weeks. Secondary outcome parameters are qualitative differences in profiles of characteristic and proving symptoms and the total number of all proving symptoms. The number of symptoms will be quantitatively analysed on an intention-to-treat basis using ANCOVA with the subject's expectation and baseline values as covariates. Content analysis according to Mayring is adapted to suit the homeopathic qualitative analysis procedure.

**Discussion:**

Homeopathic drug proving trials using the terminology of clinical trials according GCP and fulfilling current requirements for research under the current drug regulations is feasible. However, within the current regulations, homeopathic drug proving trials are classified as phase 1 trials, although their aim is not to explore the safety and pharmacological dynamics of the drug, but rather to find clinical indications according to the theory of homeopathy. To avoid bias, it is necessary that neither the subjects nor the investigators know the identity of the drug. This requires a modification to the informed consent process and blinded study materials. Because it is impossible to distinguish between adverse events and proving symptoms, both must be documented together.

**Trial registration:**

ClinicalTrials.gov identifier: NCT01061229.

## Background

Homoeopathic drug proving trials (HDP), also known as homeopathic pathogenetic trials, is one of the fundamental concepts of homeopathy and has been conducted for more than 200 years [1-4]. In addition to exposing the toxic effects of the drug, HDP serve as a key source of information for the homeopathic *materia medica*. Its purpose is to test non-toxic levels of a specific substance in healthy volunteers to determine the symptoms this substance stimulates and the types of individuals who may be sensitive to it. The profile of symptoms, recorded in an HDP by a group of healthy volunteers, serve as basis for information to find indicators of the drug in sick patients. In HDP, the drug under investigation is administered and the individual response of every volunteer to the application of the substance is described. According to the law of similars, the substance is then used to treat patients with similar symptoms. The clinical experience subsequently shapes the homeopathic drug profile.

A systematic review published in 2007 analysed HDP conducted between 1945 and 1995, showed that most HDP were of low methodological quality [1]. Most of these old HDP studies were conducted by homeopathic physicians who were mainly interested in identifying clinical indications and drug profiles for therapeutic purposes following the theory and philosophy of homeopathy. However, in recent years innovative new study methodology for HDP has been developed and tested [5-10].

Recent research [11] has focussed on two main objectives 1) using rigorous experimental designs to find out if specific effects of highly diluted homeopathic drugs compared to placebo or other controls can be identified or reproduced and 2) qualitatively identifying new symptoms and indications mainly for therapeutic purposes.

Current research in HDP focuses on re-proving old and commonly used homeopathic remedies in double-blinded and placebo-controlled trials. In these studies blinded homeopaths were asked to identify drug-specific symptoms in the diary of each study-subject and assign them to a list of potential homeopathic drugs. In the last blinded HDP conducted by this study group, two well-known homeopathic remedies (Natrium muriaticum and Arsenicum album) were tested against placebo. The blinded homeopaths identified only Arsenicum album-specific symptoms in the Arsenicum album group, only Natrium muriatricum specific symptoms in the Natrium muriaticum group and only non-specific symptoms in the placebo group. The results of this and a previous HDP showed that specific symptoms of the HDP could be identified and allocated by blinded raters with a high statistical significance [8,9,11]. To our knowledge, it is not clear whether the identification of drug-specific symptoms may also be achieved for new and unknown homeopathic drugs. Therefore, in our opinion, a key question of current HDP research is to define specific symptoms, including a list of criteria, and to develop a qualitative analysis method in order to identify them in new and unknown homeopathic drugs.

Homeopaths and researchers have developed a variety of study protocols for HDP and incorporated a variety of modern standards in the last few years [2,3,12-16].

National and European authorities require that the Guidelines for Good Clinical Practice (ICH GCP), the Declaration of Helsinki and the national drug regulations be applied for HDP and methodological and legal consequences must be considered. The European Committee for Homoeopathy (ECH) developed the "Homoeopathic drug proving guideline" [2] which for the first time, adapts traditional HDP methodology to the requirements of modern European standards. German drug regulations have meanwhile classified HDP as clinical phase 1 trials. Within this study we developed, for the first time, a study design methodology and a study-protocol that fulfils the criteria of a phase-1-trial, GCP, Declaration of Helsinki and German drug regulations and also tests its applicability and feasibility in practice. The main aim of the study is to determine whether a homeopathic drug in the potency C12 provokes more characteristic homeopathic proving symptoms after three weeks compared to a placebo in healthy volunteers. Therefore our hypotheses to test are:

H_0_: There is no difference between the number of characteristic symptoms provoked by a homeopathic drug in the potency C12 compared to placebo.

H_1_: There is a difference between the number of characteristic symptoms provoked by a homeopathic drug in the potency C12 compared to placebo.

Secondary aims are to develop and to test a qualitative analysis methodology on which to base a definition for drug-specific (characteristic) symptoms and to compile a profile of characteristic homeopathic proving symptoms of the drug being trialled for therapeutic purposes.

## Methods

### Study design

The homeopathic drug proving trial is to be conducted as a multi-centre, randomised, double-blind, placebo-controlled phase 1 trial. Subjects and investigators are not only blinded to the group allocation process but also to the identity of the drug.

### Subjects

Volunteer medical students or medical doctors are invited to take part in the trial by their investigators via email or phone.

Subjects can be included if they fulfil the following criteria: Medical doctors or medical students, over 18 years of age, who are not being treated for any acute or chronic diseases on the day of inclusion, plus written informed consent.

The following exclusion criteria apply: pregnant women or nursing mothers are excluded as are anyone who have received homeopathic treatment over the previous six weeks, or anyone who has participated in another clinical trial during the last six months, anyone with a personal or professional dependence on the study physician or sponsor as well as anyone who has been placed in hospital or other institution by authorities or decree.

### Investigators

The investigators are homeopathic medical doctors with knowledge of HDP and who have at least three years practical experience in homeopathic therapy. All investigators are required to have completed a two-day certified and standardised investigator training programme based on GCP, Declaration of Helsinki and the national drug regulations as well as specific training in the study procedures of this trial.

### Ethics and consent

All subjects will provide written informed consent prior to the inclusion in the trial. The trial will be performed in accordance with International Conference on Harmonisation Guidelines for Good Clinical Practice (ICH GCP), Helsinki Declaration and national drug regulations. Information about the trial will be provided during one-on-one interviews with the help of a written brochure for subjects. The study was approved by the Berlin Ethics Committee (Landesamt für Gesundheit und Soziales Berlin) on the 17th August 2009 (Reference: ZS EK 15, 287/09). The trial was registered under ClinicalTrials.gov: Identifier NCT01061229.

### Procedures

**Figure 1:**
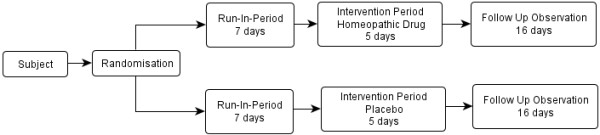
**Organisation process in homeopathic drug proving**.

The study consists of a seven day run-in period (baseline observation), a five day intervention period followed by a 16 day follow-up period (see Figure 1).

Each study centre consists of one investigator who supervises a maximum of three subjects. After giving informed consent, subjects will receive an initial physical examination, a full-length homeopathic interview of 60-120 minutes duration and training on formal and technical aspects of documentation prior to the run-in period, which is carried out by their investigator. Until now In HDP research there are no factors known to predict the outcome. The following factors are assessed at baseline: age, sex, education (medical student or doctor), history of former chronic disease, former homeopathic treatment, former participation in a homeopathic drug proving. Subjects and investigators are also asked to rate their expectations about the subjects' responsiveness to homeopathic drugs as ordinals with 4 levels, as the authors presume that this could be a factor related to the outcome.

#### Questions to subject

A. "How would you estimate your sensitivity to homeopathic remedies in general?" - possible answers: strong reaction/reaction/slight reaction/no reaction; B. "How do you expect to react to the homeopathic drug?" - answers: a very high number of symptoms/many symptoms/low number of symptoms/no symptoms.

#### Questions to investigators

A "How would you estimate your subject's sensitivity to homeopathic remedies in general?" - Response options: same as above; B: "What is your expectation about your subjects reaction to the homeopathic drug?" - response options same as above.

After starting the trial, subjects are required to document daily any new or uncommon symptoms in a semi-structured trial diary accessed through a secure internet connection. It will provide a head-to-feet structure to be filled in with free text. Every third day each subject will be contacted by phone by his or her investigator. This optimises the homeopathic quality of documentation, which is considered crucial for a successful HDT [16]. A very detailed description of symptoms experienced by the subjects is essential for the quality of the homeopathic drug proving trial. Of special importance are precise descriptions of locations, sensations, modalities, concomitant symptoms, changes in mental or emotional state. Any strange, individual, uncommon and peculiar symptoms observed should be documented as these are considered most important for the homeopathic purpose. Investigators are only permitted to read and separately comment on the documentation of their subjects. The purpose of the supervision by the investigators and the additional documentation is to highlight special symptoms, improve documentation or mark special observations as is common in HDP. Investigators are not able to change the documentation of their subjects. Their documentation is saved and stored separately.

### Randomisation and treatment allocation

**Figure 2:**
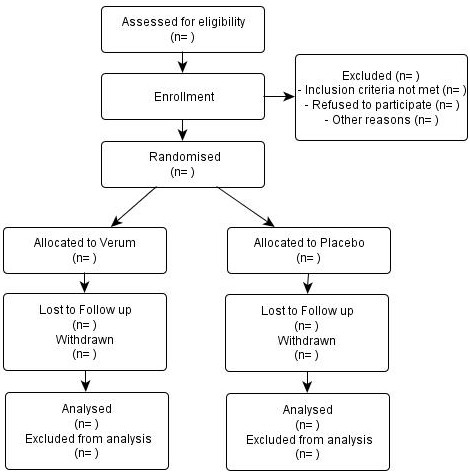
**Flow of participants through the homeopathic drug proving trial**.

Based on the "ranuni" random number generator of the SAS/STAT^® ^software, subjects will be randomly allocated to the interventions under study by block-randomisation stratified by centre (investigator). Both the centres and subjects will be coded by simple numbers unidentifiable by subjects, investigators and external persons. The random list will be sent to the Charite' central pharmacy which will prepare sealed, sequentially numbered boxes containing the study medication and send them to the centre. When a subject agrees to participate, the investigator opens the lowest numbered box and gives him/her the study medication. Each centre will keep a log file with all randomised subjects. If a subject withdraws his/her consent or discontinues to the study, he/she may not be included again. The planned flow of participants through the homeopathic drug proving trial is illustrated as flowchart in Figure 2.

### Intervention

Subjects in the intervention group are instructed to take five globules of the trial drug (potency C12), five times per day for a maximum of five days (5×5×5). The study medication is obtained from DHU (Germany), produced according to the Hahnemannian method [17]. Subjects are asked to stop taking the medication, in agreement with their investigator, if they experience any of a predefined set of proving symptoms (for definition see Table 1). Placebo consists of pure sucrose globules (DHU, Germany) that are not potentiated nor impregnated with alcohol. The administration scheme is identical in the placebo control group to that of the intervention group.

**Table 1:** Criteria for proving symptoms and characteristic symptoms

Criteria for proving symptoms	Criteria for characteristic symptoms
New symptoms: the symptom is unfamiliar and has not been observed within the last year or during the run-in-period	Characteristic symptoms are defined as proving symptoms with a strongly individualistic character:

Study participant or study physician classify the symptom as new or unusual	Symptoms affecting the whole organism of one or more study participant

The study physician classifies the symptom as new in his final evaluation	Symptoms affecting different organs or organ systems of one or more study participant

A strong aggravation or modification of present (familiar) symptoms	Symptoms accompanying a variety of other symptoms

Present familiar symptoms that have disappeared during the proving (cure)	Symptoms that occur during the trial, which appear strange, peculiar or unique to one or more study participants

	Familiar symptoms from the past or present that have been cured or strongly alleviated

### Outcome parameters

The primary outcome parameter is the number of characteristic proving symptoms per subject, derived from the qualitative data analysis of the homeopathic proving drug compared to placebo within three weeks after the initial dose of the drug or the placebo is administered. Definitions of characteristic symptoms are given in Table 1.

Secondary outcome parameters are: the total number of proving symptoms irrespective of whether they are characteristic or not (definition of proving symptoms see Table 1) and the number of serious adverse events.

Qualitative differences in the profiles of characteristic proving symptoms from the homeopathic drug and the placebo will also be compared and the inter-coder reliability of the qualitative evaluation of characteristic proving symptoms will be calculated.

### Sample size calculation

It is planned to include 30 healthy volunteers. Assuming a drop out rate of 20%, 24 subjects will be available for analysis. In this study, an unpaired t-test with a two-sided level of 5% has a power of 80% to detect a group difference of 20 ± 4 vs 15 ± 4 (mean ± standard deviation) characteristic proving symptoms.

### Analysis

The trial diaries with the data entries of subjects and investigators provide the data for the qualitative and quantitative analysis. The qualitative analysis will be carried by experienced homeopathic doctors trained in qualitative research methodology, the quantitative analysis by a statistician.

## Qualitative analysis

Data are coded by content analysis according to Mayring [18] and analysed using Atlas.ti 5.2 software. Homeopathic definitions for proving symptoms and characteristic symptoms (Tables 1 and 2) serve as predefined categories for the coding processes.

**Table 2:** Coding guideline for the homeopathic drug proving

Categories +Subcategories	Definition	Example	Code (Example)
**Locality**	Anatomic location where the symptom appears.	Nose, chest.	**QL**
+ specification			+ QL-nose
			+ QL-chest
**Sensation**	Sensation that is observed in a symptom.	Stitch, dryness.	**QS**
+ specification			+QS-stitch
			+QS-dryness
**Modality**	Factors which aggravate (agg) or ameliorating (amel) a symptom	Aggravation by cold wind, amelioration with warmth.	**QM**
+ specification			+QM-agg-cold-wind
			+QM-amel-warmth
**Concomitant**	Symptoms that typically accompany other symptoms.	Headaches accompany anticipatory anxieties.	**QC**
+ specification			+QC-headaches
**Times**	Times when symptoms appear or disappear	Headaches at 7 am.	**QT**
+ specification			+QT-7am
**Mind**	Mental or emotional disturbances.	Fear, sadness	**QM**
+ specification			+QM-fear
			+QM-sadness
**Clinical finding**	Clinical findings and signs.	Eczema, sweating	**QF**
+ specification			+QM-eczema
			+QM-sweating

Q = Quality; L = Locality; S = Sensation; M = Modality; C = Concomitant; T = Times; M = Mind; F = Clinical findings; agg = Aggravation; amel = Amelioration

**Figure 3:**
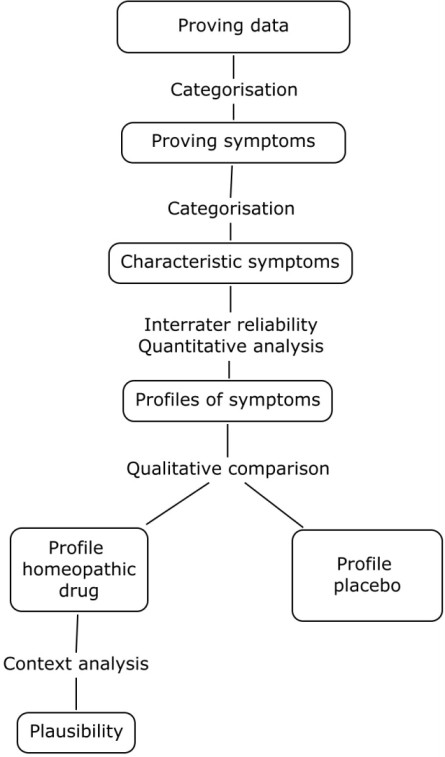
**Data analysis process in homeopathic drug proving trial**.

The qualitative data analysis consists of three steps: categorisation of proving symptoms, categorisation of characteristic symptoms, and context analysis (Figure 3).

After closing the trial, the investigators obtain the complete body of text from the trial diary for each of their subjects and are asked to categorise "proving symptoms" in accordance to the prescribed definitions. Subsequently, a second investigator reviews coding and categorisation. In cases of contradictory categorisations, a review committee of two investigators will decide on the categorisation. At the end of this process, a list of proving symptoms for each subject will be compiled.

The categorisation of characteristic symptoms is the next step. To identify characteristic symptoms which occurred in more than one subject, an inter-subject comparison of each study arm is needed. However, investigators will still be blinded from the treatment allocation. This analysis is performed independently by two experienced investigators. The inter-coder reliability is calculated by the study statistician. The data is further structured into the following categories: localities, sensations, modalities and concomitants, times, mind and clinical findings.

Profiles of characteristic symptoms are compiled for both groups which can now be compared qualitatively.

Context analysis is the final part and will be conducted when the drug identity has been revealed at the end of the trial. The results will be related to the traditional and scientific knowledge available on the drug being investigated and discussed in the research team. The aim is to find out whether the data fits into the body of knowledge already available on the particular drug and its plausibility.

## Quantitative analysis

The main outcome will be analysed on an intention-to-treat basis by univariate analyses of covariance (ANCOVA) which includes the group (two levels), subject's expectations (ordinal with 4 levels) and the respective baseline value (linear) as covariates. From these model we will estimate the baseline adjusted treatment effect and its 95% confidence interval (CI). The reported *P*-value will be based on a two-sided t-test within this model, and a *P *< 0.05 will be considered significant. Missing values will be multiply imputed according to Rubin's suggestions [19]. In detail, 20 multiple copies of the original data set will be generated, replacing missing values by a randomly generated value according to the MCMC algorithm. Each copy will be analysed as a complete data set with the abovementioned ANCOVA model and the results will be combined appropriately. Sensitivity analyses will include some extensions of the statistical model, in this way modelling the centre as a random factor and adding the center's expectation (ordinal with 4 levels) as an additional fixed factor.

Analyses of the secondary outcome parameters will rely on the same statistical models, hereby replacing the baseline value of the main outcome parameters by the baseline value of the parameter under consideration.

All analyses will be performed using SAS/STAT^®^.

## Discussion

This study transfers the traditional concept of homeopathic drug proving trials into current clinical drug trial methodology and standards and adapts it for the guidelines of the drug regulations for phase 1 trials. A standardised qualitative analysis procedure is combined with quantitative analysis techniques. So far, our experience suggests that designing homeopathic drug proving trials using the terminology of clinical trials according GCP and fulfilling the current requirements for research under the national drug regulations is feasible. However, there are some aspects differing from conventional standards that need to be discussed:

## Classification as phase 1 trial

According to the German drug regulations, a homeopathic drug proving trial is a phase 1 trial. The objective of a conventional phase 1 trial is to assess the safety and to gain knowledge about their pharmacological dynamics in a small number of subjects. In contrast, the aim of a homeopathic drug proving trial is to identify new symptom patterns to identify therapeutic indications according to the law of similars and enhance the homeopathic knowledge and *materia medica *[20]. The subjects are usually doctors or students trained in homeopathy and not simply healthy volunteers.

## Informed consent and blinding

Another aspect is that in conventional trials it is necessary to inform subjects and investigators about the identity of the drug, the risks and potential benefits. In an HDP this has to be hidden from the subjects and investigators to avoid bias in detecting and reporting symptoms. After lengthy discussions regarding our study, the Berlin ethics commission and the German health authorities agreed on keeping the study blinded for the subjects and investigators, but insisted on reporting the identity of the trialled substance, its risks and potential benefits to the authorities. This makes the whole procedure more complicated, because most materials (e.g. study protocol, investigators brochure) have to be produced in a blinded and a non-blinded version. We decided that only two members of the study team (the principal investigator and one study nurse) would know the identity of the drug to minimize the risk of involuntarily disclosing the drug on trial.

## Preclinical toxicological tests

In drug trials preclinical toxicological data are considered necessary before testing the substance in human subjects. The risk of a substantial intoxication in homeopathic drugs of a high potency is very low since the compound is very dilute. The German drug agency advises against preclinical toxicological tests in potencies from C12 (10^-24^) [21]. In our trial we will follow this recommendation and use C12 potency.

## Classification of Adverse Events

At present it is impossible to distinguish adverse events from homeopathic proving symptoms due to a lack of knowledge and subsequent criteria. In our homeopathic drug proving trial, all symptoms are to be recorded in an online diary by subjects and investigators. Severe adverse events are to be separately documented and reported to the sponsor, the ethical commission and the health authorities, following the current regulations.

## Qualitative analysis

In the homeopathic community there is no consensus to date on how to qualitatively analyse the trial data which consists of large bodies of text. Criteria defining proving symptoms exist, but it is unclear how to analyse them and how reliable these criteria are. In this paper we suggest the use of content analysis, according to Mayring [18], to analyse the texts, because it fulfils five basic criteria: 1. the opportunity to categorise, 2. a combination of fixed and open categorisation, 3. the applicability to the homeopathic terminology, 4. the possibility to use software and 5. the possibility to reproduce the analysis. However, this method is used for homeopathic purposes and it is unclear whether the chosen methodology will be fully suitable. Indeed, this is a very crucial point: The qualitative analysis is necessary following any quantitative analysis. If it fails, all statistical results (including p-values), are potentially biased. Therefore, we will test for reliability of the coding of the primary outcome parameter characteristic proving symptoms, which will be performed independently by two experienced homeopaths. Therefore the results are also dependent on the experience of the coding raters.

To optimally fulfil homeopathic criteria according to Hahnemanns Organon (§143), we will use characteristic proving symptoms as the primary outcome parameter. We feel that this aligns with the homeopathic philosophy of emphasizing the superior role of individualistic and peculiar symptoms.

## Competing interests

The authors declare that they have no competing interests.

## Authors' contributions

MT is research coordinator, designed the trial, wrote the draft of this manuscript and obtained the main trial funding. UH co-designed the trial, coordinates the practical study organisation and monitors the trial. JD and CS co-developed the study design. RL provided statistical advice and wrote the relevant sections of the protocol. HA chose the homeopathic drug. CW is principal investigator, conceived the project, co-led the design and coordination of the trial, edited the manuscript and has the overall scientific responsibility. All authors read and approved the final manuscript.
